# C/N-Dependent Element Bioconversion Efficiency and Antimicrobial Protein Expression in Food Waste Treatment by Black Soldier Fly Larvae

**DOI:** 10.3390/ijms23095036

**Published:** 2022-05-01

**Authors:** Ning Jin, Yanxia Liu, Shouyu Zhang, Shibo Sun, Minghuo Wu, Xiaoying Dong, Huiyan Tong, Jianqiang Xu, Hao Zhou, Shui Guan, Weiping Xu

**Affiliations:** 1 School of Ocean Science and Technology & Panjin Institute of Industrial Technology, Dalian University of Technology, Panjin Campus, Panjin 124221, China; jinningdut@163.com (N.J.); yanxia@mail.dlut.edu.cn (Y.L.); zshouyu@mail.dlut.edu.cn (S.Z.); wumh@dlut.edu.cn (M.W.); dongxy@dlut.edu.cn (X.D.); tonghuiyan@dlut.edu.cn (H.T.); zhouhao@dlut.edu.cn (H.Z.); 2 School of Life Science and Pharmaceutical Sciences, Dalian University of Technology, Panjin Campus, Panjin 124221, China; sunshibo@mail.dlut.edu.cn (S.S.); jianqiang.xu@dlut.edu.cn (J.X.); 3 Key Laboratory of Industrial Ecology and Environmental Engineering, Ministry of Education China, Dalian University of Technology, Dalian 116024, China; 4 State Key Laboratory of Fine Chemicals, Dalian R&D Center for Stem Cell and Tissue Engineering, School of Chemical Engineering, Dalian University of Technology, Dalian 116024, China; guanshui@dlut.edu.cn

**Keywords:** black soldier fly larvae, food waste, C/N ratio, antimicrobial proteins, transcriptome, *Hermetia illucens*, element recycle, gene family

## Abstract

The black soldier fly (BSF), *Hermetia illucens*, has emerged as a promising species for waste bioconversion and source of antimicrobial proteins (AMPs). However, there is a scarcity of research on the element transformation efficiency and molecular characterization of AMPs derived from waste management. Here, food waste treatment was performed using BSF larvae (BSFL) in a C/N ratio of 21:1–10:1, with a focus on the C/N-dependent element bioconversion, AMP antimicrobial activity, and transcriptome profiling. The C-larvae transformation rates were found to be similar among C/Ns (27.0–35.5%, *p* = 0.109), while the N-larvae rates were different (*p* = 0.001), with C/N 21:1–16:1 (63.5–75.0%) being higher than C/N 14:1–10:1 (35.0–45.7%). The C/N ratio did not alter the antimicrobial spectrum of AMPs, but did affect the activities, with C/N 21:1 being significantly lower than C/N 18:1–10:1. The lysozyme genes were found to be significantly more highly expressed than the cecropin, defensin, and attacin genes in the AMP gene family. Out of 51 lysozyme genes, C/N 18:1 and C/N 16:1 up-regulated (*p* < 0.05) 14 and 12 genes compared with C/N 21:1, respectively, corresponding to the higher activity of AMPs. Overall, the element bioconversion efficiency and AMP expression can be enhanced through C/N ratio manipulation, and the C/N-dependent transcriptome regulation is the driving force of the AMP difference.

## 1. Introduction

Global food waste production reached 931 million tons per year as estimated by the United Nations Environmental Programme in 2021 [1]. Food waste is a big source of greenhouse gas emission and a significant burden to waste management systems while exacerbating food insecurity [1]. As such, the Sustainable Development Goal 12.3 was implemented with the aim of halving food waste by 2030 [2]. Conversional technology for food waste valorization includes composting and anerobic digestion [3,4], and recent advances were made in the synthesis of biodiesel [5,6,7,8], the pyrolysis treatment associated with biochar application [9,10,11], and the passivation of heavy metals [12,13]. Bioremediation using insect biomass also emerged as an innovative alternative to the conventional methods [3]. The black soldier fly (BSF), *Hermetia illucens* L., has become the optimal species for insect mass raring and industrial application owing to the significant advantages thereof, such as voracious feeding behavior, short generation times, and rapid accumulation of insect biomass [14,15].

Compared with open-air composting, BSFL treatment can reduce over 90% of CO_2_ and 94% of NH_3_ emission, generating considerable benefits for greenhouse gas reduction and odor control [16,17,18,19,20]. In contrast to greenhouse gas reduction, the C and N conversion rates by BSFL were found to peak at 13% for the C-larvae bioconversion ratio (substrate C ratio obtained by larvae) and 28% for the N-larvae bioconversion ratio (substrate N ratio obtained by larvae) [19,20,21,22]. Evidently, improving the C-larvae and N-larvae conversion rates would elevate the element recycle efficiency and improve the sustainable trait of BSFL treatment. Among the physiochemical properties of BSFL treatment, C/N could be a crucial parameter that interacts with the element transformation efficiency. In previous studies, C/N 25:1, 15:1, and 14:1 were found to be optimal for either C or N transformation in the BSFL treatment of pig manure [20], brewer’s spent grain [21], and landfill leachate [22]; however, the optimal C/N ratio for food waste has not been investigated, nor has the C/N impact on the element bioconversion efficiency. Further, in the aforementioned studies, materials with a low C/N ratio (<11:1) were utilized, and the C/N ratio was elevated with fiber-based materials for the C/N ratio adjustment. In contrast, the food waste collected from canteens or restaurants was found to be typically rich in starch with C/N ratios > 20:1 [23]. Rather than abundance, nitrogen deficiency might be the problem; therefore, supplying a source of N for the C/N adjustment could be more reliable and practical for the improvement of element bioconversion efficiency in the BSFL treatment.

Aside from the ability to upcycle organic waste streams, the BSFL also shows promise as a source of antimicrobials [24]. The antimicrobial proteins (AMPs) extracted from the BSFL illustrated an inhibition effect against a wide spectrum of pathogens in vitro and mice in vivo, for example, *Salmonella* spp., *Staphylococcus aureus*, and *Klebsiella pneumonia* [25,26,27,28,29]. However, for most of the aforementioned findings, the BSFL was reared in clean artificial substances, and often integrated with needling and bacterial injection for the stimulation of AMPs [25,26,29]. The BSFL derived from waste management, especially originating from food waste treatment without specific physical/biological stimulation, has not been investigated.

Additionally, the focus of previous studies was largely on the sequence, recombinant expression, and/or activity analysis of a single AMP [24,28,30]. As such, there is a scarcity of research on the entire AMP gene family and the expression characterization of AMPs following waste treatment. Next generation sequencing (NGS) technology can be beneficial in closing the research gap. Up to date, although five transcriptome studies of the BSFL were published [31,32,33,34,35], only Vogel et al. (2018) investigated the expression profile of the AMP gene family. However, the AMP expression level was not found to be correlated with the antimicrobial activity [31], which was probably attributed to less accuracy of transcriptome de novo analysis compared with reference genome-based analysis. Fortunately, Zhan et al. (2020) released the first BSF genome sequencing data (NCBI Bioproject No. PRJNA547968) and found that the BSF had the largest AMP gene family in insects [36]. Moreover, Generalovic et al. (2021) released an improved chromosome-scale assembly of the BSF genome (NCBI Bioproject No. PRJEB37575) with 16,478 protein-coding genes annotated, among which 36 cecropin, 4 attacin, 12 defensin, and 54 lysozyme genes were found in the AMP gene family [37]. A high-quality reference genome would facilitate the accuracy of transcriptome analysis, and there is significant research interest in analyzing the BSFL transcriptome based on the recently updated genome, and further characterizing the AMP antimicrobial activity with the transcriptome traits of the AMP gene family.

Overall, the aim of the present study was to investigate the C/N-dependent element transformation, AMP antimicrobial activity, and transcriptome traits of the AMP gene family, in order to generate feasible tools for improved element recycle efficiency and enhanced AMP expression by the BSFL during food waste treatment.

## 2. Results

### 2.1. Bioconversion Performance and AMP Extraction Rate

**Figure 1:**
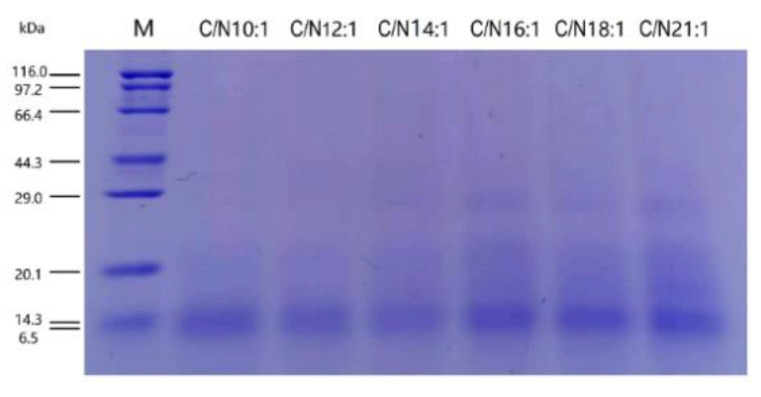
SDS-PAGE analysis of the resolved lyophilized AMP extracts.

There were significant differences in the waste reduction (WR) efficiency among C/Ns (Table 1), and the C/N 21:1, 18:1, and 16:1 groups were higher than those of the C/N 14:1, 12:1, and 10:1 groups. However, the larvae yields (LY) were similar among C/Ns. Both the concentration and extraction rate (ER) of crude AMPs were not different among C/Ns (Table 1). The SDS-PAGE analysis showed that the resolved lyophilized AMPs were proteins with a molecular weight ranging from 0 to 44.3 kDa (Figure 1). What’s more, several strong bands were observed near 29.0 kDa and 14.3 kDa (Figure 1).

**Figure 2:**
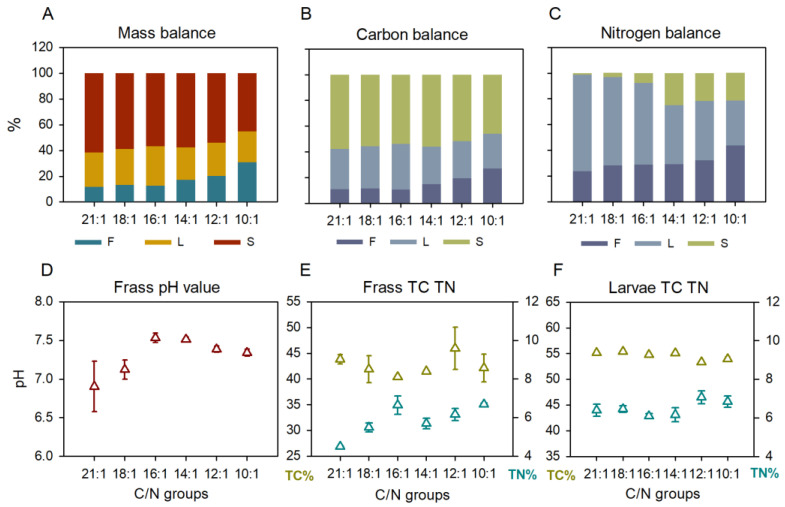
The mass, carbon and nitrogen balance and the pH, TC, and TN properties after 10 d of food waste treatment. F, frass; L, larvae; S, gaseous loss. Data points were presented as means (**A**–**C**) or mean ± SD (**D**–**F**), n = 3.

The mass balance showed the same trends as the WR and LY results (Figure 2A). Compared with the C/N 21:1, 18:1, and 16:1, the C/N 14:1, 12:1, and 10:1 exhibited higher ratios of frass remained (*p* < 0.001), similar ratios of larvae produced (*p* < 0.177), and lower ratios of gaseous loss (*p* < 0.001). The carbon balance resulted in a similar pattern as the mass balance with limited differences in the C-larvae conversion rates (*p* = 0.109, Figure 2B). The C-larvae rates were 31.1 ± 1.3% (21:1), 32.7 ± 3.8% (18:1), 35.5 ± 1.5% (16:1), 29.2 ± 4.9% (14:1), 28.7 ± 2.3% (12:1), 27.0 ± 0.9% (10:1), respectively, and the C-frass rates (substrate C ratio remained in frass) were 11.3 ± 1.5% (21:1), 11.9 ± 0.9% (18:1), 11.0 ± 0.7% (16:1), 15.2 ± 1.0% (14:1), 19.8 ± 2.6% (12:1), 27.3 ± 3.6% (10:1), respectively. The nitrogen balance differed among C/Ns, with trends of a decreasing N-larvae rate (*p* < 0.001) and increasing N-frass rate (substrate N ratio remained in frass) (*p* = 0.001) across C/N 21:1 to C/N 10:1 (Figure 2C). The N-larvae rates were 75.0 ± 7.0% (21:1), 68.6 ± 7.5% (18:1), 63.5 ± 3.2% (16:1), 45.7 ± 6.1% (14:1), 46.2 ± 4.2% (12:1), 34.9 ± 0.9% (10:1), respectively, and the N-frass rates were 24.1 ± 3.0% (21:1), 28.4 ± 4.6% (18:1), 29.2 ± 3.9% (16:1), 29.5 ± 1.8% (14:1), 32.3 ± 3.0% (12:1), 43.9 ± 2.6% (10:1), respectively. The significant drop in the N-larvae rate occurred in between C/N 16:1 and C/N 14:1 (Figure 2C).

The frass pH value also varied among C/Ns (*p* = 0.007), and C/N 21:1 showed a lower value (pH 6.9 ± 0.3) than the other C/Ns (pH 7.1–7.5) (Figure 2D). The frass total carbon (TC, 40.4–45.9%) did not differ among C/Ns (*p* = 0.266), whereas the frass total nitrogen (TN, 4.5–6.7%) was distinguished (*p* < 0.001) with C/N 21:1 (TN 4.5 ± 0.1%) lower than the other C/Ns (TN 5.5–6.7%) (Figure 2E). The larval TC was different among C/Ns (*p* < 0.001) with C/N 12:1 and 10:1 (53.3–53.9%) lower than C/N 21:1–14:1 (54.8–55.4%), and the larval TN was also different (*p* = 0.032) with C/N 16:1 (6.1 ± 0.1%) lower than C/N 12:1 (7.1 ± 0.3%) (Figure 2F).

### 2.2. Inhibition Concentrations Assay

**Figure 3:**
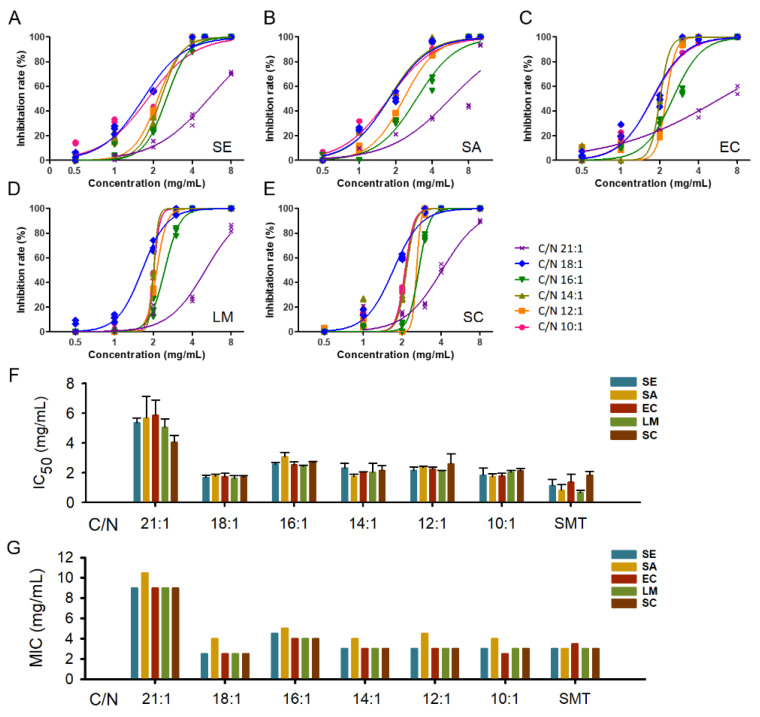
The half and minimum inhibition concentration assay of the crude AMP extracts and the antibiotic control sulfamonomethoxine. (**A**–**E**), the inhibition curves of five pathogenic strains by AMP extracts; (**F**), the estimated IC_50_ and 95% CI; G, the MIC results. SE, *Salmonella enterica* serovar typhimurium; SA, *Staphylococcus aureus*; EC, *Escherichia coli O157:H7*; LM, *Listeria Monocytogenes;* SC, *Shigella Castellani*; SMT, sulfamonomethoxine.

The half inhibition concentration (IC_50_) and minimum inhibition concentration (MIC) assay were carried out corresponding to the five pathogenic strains and illustrated in Figure 3. The IC_50_ values of AMP extracts varied in 1.62–5.88 mg/mL (Figure 3A–E), and the difference mainly existed among C/Ns whereas not across strains (Figure 3F). The C/N 21:1 showed significantly higher IC_50_ values (4.07–5.88 mg/mL) than the other C/Ns, and C/N 18:1 obtained lower IC_50_ values (1.62–1.77 mg/mL) than C/N 16:1–10:1 (1.75–3.09 mg/mL). The antibiotic sulfamonomethoxine (SMT) showed IC_50_ values (0.65–1.82 mg/mL) similar to C/N 18:1 (Figure 3F). The MIC test exhibited a similar trend as the IC_50_ assay (Figure 3G). C/N 21:1 obtained higher MIC values (9.0–10.5 mg/mL) than C/N 18:1 (MIC 2.5–4.0 mg/mL), C/N 16:1 (MIC 4.0–5.0 mg/mL), C/N 14:1 (MIC 3.0–4.0 mg/mL), C/N 12:1 (MIC 3.0–4.5), and C/N 10:1 (MIC 2.5–4.0 mg/mL). The SMT showed MIC values of 3.0–3.5 mg/mL, similar to C/N 18:1–10:1 (Figure 3G). Among pathogenic strains, the *Staphylococcus aureus* (SA) strain exhibited higher MIC values than the other strains across C/Ns (Figure 3G).

### 2.3. Inhibition Zone Assay

**Figure 4:**
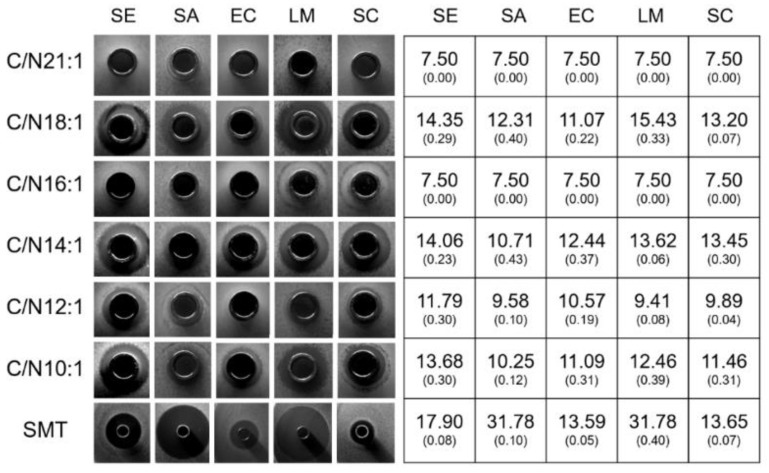
The inhibition zone assay of the crude AMP extracts and the antibiotic control sulfamonomethoxine. Diameters of inhibition zone (DIZ) were illustrated in the table with large numbers representing the mean value and small numbers representing the standard deviation (n = 3). SE, *Salmonella enterica* serovar typhimurium; SA, *Staphylococcus aureus*; EC, *Escherichia coli O157:H7*; LM, *Listeria Monocytogenes;* and SC, *Shigella Castellani*; SMT, sulfamonomethoxine.

Inhibition zones were observed in C/N 18:1, 14:1, 12:1, and 10:1, whereas not in C/N 21:1 and 16:1 (Figure 4), suggesting the working concentration of 10 mg/mL AMPs might not be sufficient for C/N 21:1 and 16:1. However, no bacteria were found growing inside the Oxford cups for C/N 21:1 and 16:1 (Figure 4). Among C/N 18:1, 14:1, 12:1, and 10:1, C/N18:1 and 14:1 showed greater diameters of inhibition zones (DIZs) than C/N 12:1 and 10:1 (Figure 4). Among the pathogenic strains, the SE, LM, and SC exhibited higher DIZs than the SA and EC (Figure 4). The antibiotic SMT exhibited greater DIZs than the AMP extracts across C/Ns (Figure 4).

### 2.4. Transcriptome Analysis

**Figure 5:**
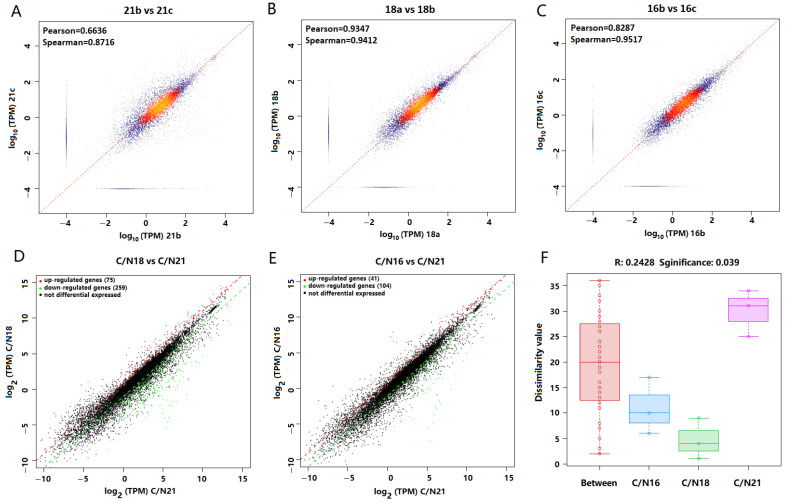
The transcriptome comparison in terms of TPM abundance between replicates and among C/N 21:1, 18:1, and 16:1. (**A**–**C**), the correlation analysis between replicates. (**D**,**E**), the comparison analysis of C/N 18:1 vs. 21:1 (**D**) and C/N 16:1 vs. 21:1 (**E**) with statistical significance defined as *q* < 0.05 and log_2_ fold change >1 (red dots) or <−1 (green dots). (**F**), the comparison of entire transcriptomes with anosim analysis.

The transcriptome reference-based assembly identified transcripts of 15,557 genes within 16,478 protein-coding genes in the reference genome PRJEB37575. The transcript abundance ranged from 10^−4^ to 10^4^ TPM with the majority varied in 10^−2^–10^3^ TPM (Figure 5A–C). The replicate samples exhibited significant correlations (*r* = 0.871–0.952) in terms of TPM abundance (Figure 5A–C), suggesting good quality and reproducibility of transcriptome reference-based analysis. The overall transcriptome comparison revealed that C/N 21:1 was significantly different from C/N 18:1 and C/N 16:1 (*p* = 0.039), while C/N 18:1 and C/N 16:1 were similar (Figure 5F). There were more genes up-regulated than those down-regulated in the comparison of both C/N 18:1 vs. C/N 21:1 and C/N 16:1 vs. C/N 21:1 (Figure 5D,E).

**Figure 6:**
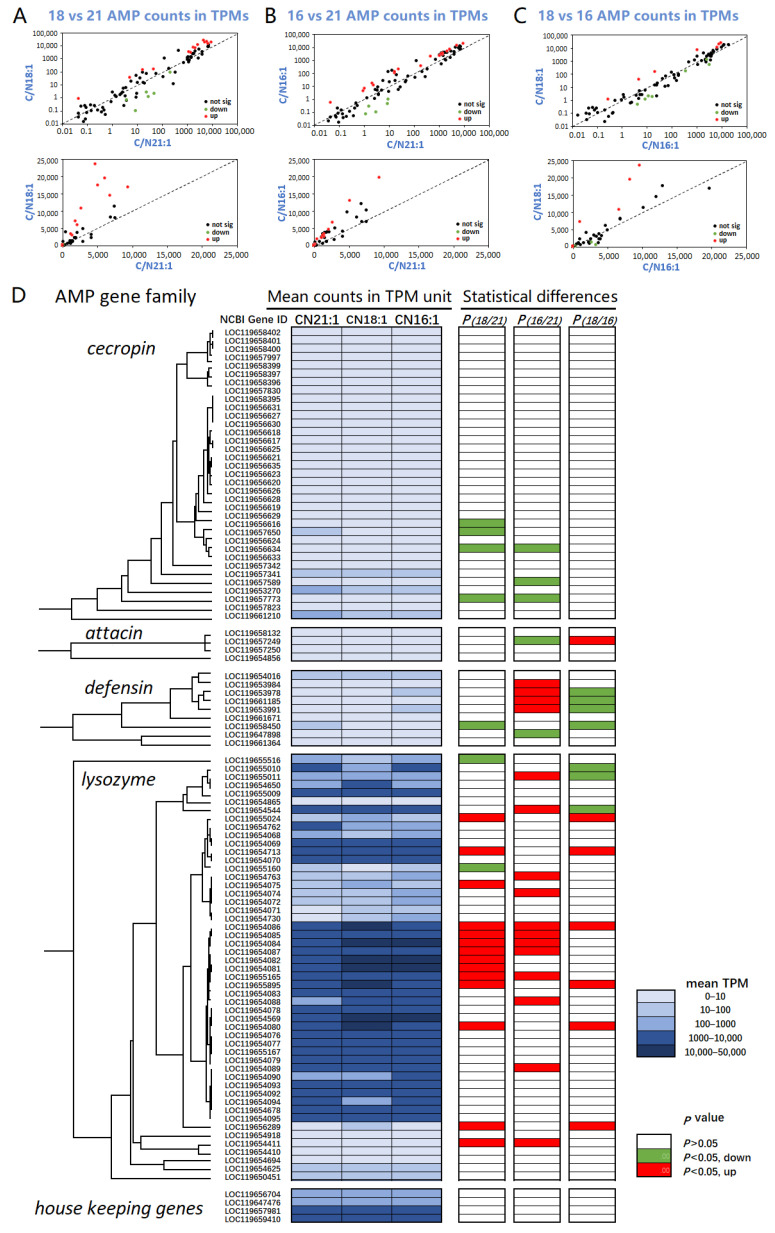
The transcriptome profiling of AMP gene family among larvae reared in the C/N 21:1, 18:1, and 16:1 conditions. (**A**–**C**), the correlations of transcript abundance in terms of TPMs; axes were represented in log scale and linear scale, respectively; significantly regulated genes (*p* < 0.05) were marked in red (up) or green (down) color. (**D**), the expression analysis of the AMP gene family; evolutionary trees of AMP gene family were presented; average expression levels in TPM units were displayed in blue color scale, and statistical differences of C/N 18:1 vs. 21:1, C/N 16:1 vs. 21:1, and C/N 18:1 vs. 16:1 were shown in the red (up-regulated) or green (down-regulated) color, being consistent with the dot colors in the graph (**A**–**C**).

Among the 36 cecropin, 4 attacin, 12 defensin, and 54 lysozyme genes in the BSF genome, transcripts of 35 cecropin, 4 attacin, 9 defensin, and 51 lysozyme genes were identified in the current transcriptome, and their gene ID were listed in the Figure 6. The transcriptome analysis of the AMP gene family revealed that the lysozyme genes expressed at higher levels than the cecropin, defensin, and attacin genes regardless of C/Ns (Figure 6). A total of 29 out of 51 lysozyme genes were expressed at the 10^3^–10^5^ TPM level, while cecropin and defensin genes expressed at 0–10^2^ TPM level, and attacin genes expressed at the 0–10^0^ TPM level (Figure 6D). In the linear scale comparison of AMP transcripts, only lysozyme genes could be shown, while the log scale plot exhibited all four types of AMP genes (Figure 6A–C). Compared with C/N 21:1, C/N18:1 and C/N16:1 significantly up-regulated 14 and 12 out of 51 lysozyme genes, respectively, and down-regulated a few cecropin, defensin, and attacin genes (Figure 6D). Focusing on lysozyme genes, the expression level ranked as C/N 18:1 > C/N 16:1 > C/N 12:1 (Figure 6D). In the lysozyme evolution tree, the branch of LOC119654086–LOC119654095 genes exhibited a higher expressing level than the other branches (Figure 6D). The exact TPM data presented in Figure 6 can be obtained at https://doi.org/10.6084/m9.figshare.17194964.v1 (accessed on 18 March 2022).

**Figure 7:**
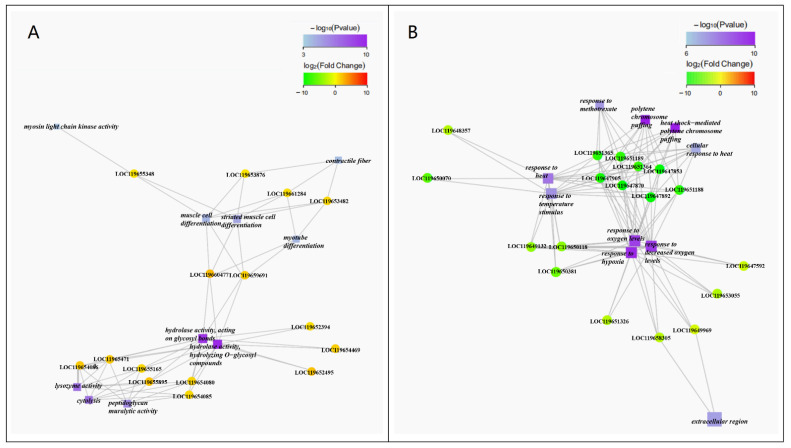
The GO enrichment network of significantly up-regulated (**A**) and down-regulated (**B**) gene functions and genes in the transcriptome comparison of C/N 18:1 vs. C/N 21:1. The blue-purple squares represent significantly regulated gene functions with the color correlated with the *p* value, and the green-yellow-red circles represent significantly regulated gene ID numbers with the color correlated with the fold changes.

Due to the similarity of the transcriptome between C/N 18:1 and C/N 16:1 (Figure 5F), the GO enrichment network focused on the comparison of C/N 18:1 and C/N 21:1 (Figure 7). Gene functions related with hydrolase activity (acting on glycosyl bonds), cytolysis (lysozyme activity), and muscle cell differentiation were found significantly up-regulated (Figure 7A). Gene functions related with response to heat or oxygen levels and polytene chromosome puffing were found significantly down-regulated (Figure 7B).

**Figure 8:**
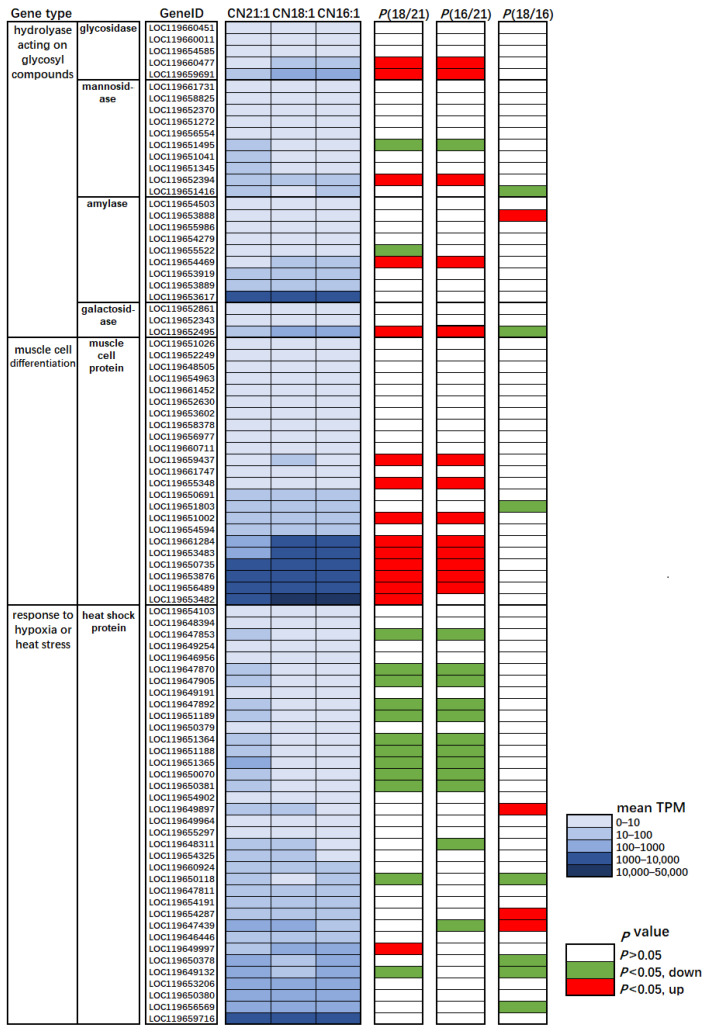
The transcriptome profiling of glycosyl hydrolase, muscle protein, and heat shock protein gene families among larvae reared in the C/N 21:1, 18:1, and 16:1. The average TPMs (n = 3) are displayed in blue color scale, and the statistical differences of C/N 18:1 vs. 21:1, C/N 16:1 vs. 21:1, and C/N 18:1 vs. 16:1 are shown in the red (up-regulated) or green (down-regulated) color.

To confirm the results of the GO enrichment network further, three typical gene families, i.e., hydrolase acting on glycosyl compounds, muscle cell proteins, and heat shock proteins, were searched in the transcriptome profile and compared for their TPM abundance. Compared with C/N 21:1, C/N 18:1 and C/N 16:1 up-regulated (*p* < 0.05) 5 out of 27 hydrolase genes and 8–9 out of 23 muscle cell protein genes, while down-regulating (*p* < 0.05) 12 out of 36 heat shock protein genes (Figure 8). The exact TPM data presented in Figure 8 could be obtained at https://doi.org/10.6084/m9.figshare.17194964.v1 (accessed on 18 March 2022).

### 2.5. Bacterial community analysis

**Figure 9:**
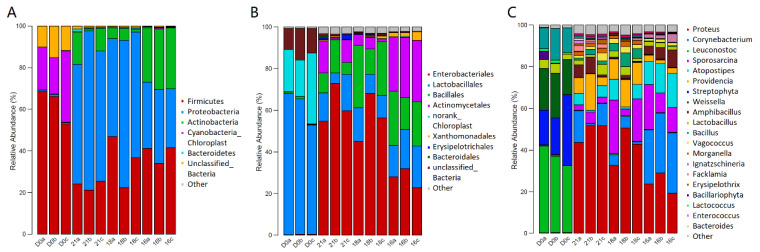
The bacterial community of food waste (d 0) and larval frass (d 10). A, the phylum level; B, the order level; and C, the genus level. D0, food waste; 21, frass of C/N21:1; 18, frass of C/N18:1; and 16, frass of C/N16:1. Triplicate samples were illustrated as the a, b and c, respectively.

The bacterial community analyses were performed for the food waste (d 0) and C/N21:1, C/N18:1, and C/N16:1 frass sample (d 10) (Figure 9). The food waste was mainly composed by the genus of *Leuconostoc, Streptophyta,* and *Weissella* spp., which was significantly different from the frass samples on all the phylum, order and genus levels (Figure 9). The frass samples were occupied by the genus of *Proteus*, *Corynebacterium*, and *Sporosarcina* spp. (Figure 9C). However, the frass of C/N21:1, C/N18:1, and C/N16:1 was not distinguished for their bacterial community on either the phylum (*P* = 0.5), order (*P* = 0.2) or genus (*P* = 0.1) level (Figure 9).

## 3. Discussion

### 3.1. The Element Bioconversion Efficiency

Based on the C and N balance analysis, the C-larvae conversion rate reached 27.0–35.5%, and the N-larvae rate achieved 35.0–75.0% in the present study, regardless of the C/N ratio difference. Compared with the rates found in previous studies, that is 2.0–13.4% for the C-larvae rates and 9–28% for the N-larvae rates [19,20,21,22], the present C-larvae and N-larvae bioconversion rates exhibited significant improvement. Regarding the C conversion performance, the improvement in C-larvae rates could be highly attributed to the energy density of food waste, highly digestible C source, and feasible larvae/waste (L/W) ratio, but less attributed to the substrate C/N ratio, since the C-larvae rates among C/Ns were not different (*p* = 0.109). The present food waste collected from canteens possessed higher energy [38] than the waste used in previous studies, such as animal manure, brewer’s spent grain, abattoir waste, and landfill leachate, which may have contributed to the high C-larvae conversion rate. Further, fiber-based materials, such as sawdust and straw, were not utilized in the present treatment for the C/N adjustment, and the food waste C could be highly digestible to the BSFL compared with fiber-based materials, which also contributed to the high C-larvae conversion performance. Finally, the L/W ratio in the present study might have been beneficial to the C-larvae bioconversion. In the present study, 1200 6-d-old larvae were used as 500 g food waste with a L/W ratio of 2.4:1. In previous studies, Lalander et al. (2019) used a L/W ratio of 0.4:1–1.2:1 [39]; Grossule et al.(2020) used a L/W ratio of 0.5:1 [22]; Beesigamukama et al. (2021) used a L/W ratio of 0.3:1 [21]; and Pang et al. (2020a, b) used a L/W ratio of 1.5:1–1.6:1 [19,20]. Since the L/W ratio interacts with waste bioconversion efficiency, a higher L/W ratio than in previous studies was adopted in the present study, which may have contributed to the C-larvae bioconversion. Further investigations should be conducted to clarify the potential interaction between the L/W ratio and the element recycling performance.

Although the C/N ratio did not affect the C-larvae conversion rates, the C-frass and C-loss rates were influenced, in that a C/N ratio < 14:1 resulted in a significant increase in the C-frass rates, and a C/N ratio 10:1 led to a significant decrease in C-loss. Such findings suggest that over-tuning of the C/N ratio to a level of < 14:1 may result in a negative effect of waste C consumption and should be avoided, which is consistent with previous findings of the present authors [23].

In contrast to the C-larvae bioconversion rate, the N-larvae conversion performance was significantly affected by the C/N ratio. C/N 21:1–16:1 was optimal for N-larvae conversion rates, while C/N 14:1–10:1 resulted in N-loss in gaseous form (Figure 2C). C/N 21:1, 18:1, and 16:1 resulted in N-larvae conversion rates of 75%, 69%, and 64%, respectively, suggesting that the food waste substrate could be highly deficient for N supply to the BSFL, and that > 64% of waste N was transformed into larval biomass. Further, C/N ratios 14:1–10:1 resulted in a negative effect on N-loss, suggesting that over supply of the N source was not necessary. Since the N-loss was positively correlated with the frass pH status, changing the environmental pH from acid to alkaline status may result in N-loss in ammonia form [19]. C/N 14:1–10:1 exhibited a frass pH of 7.3–7.5, which was relatively higher than the pH 6.9–7.3 of C/N 21:1–16:1. As such, C/N 14:1–10:1 may result in higher pH conditions or the earlier changing of acid to alkaline status than C/N 21:1–16:1, which led to N-loss in the ammonia form.

Regarding the C/N optimization for the N-larvae rate improvement, Beesigamubama et al. (2021) found that the optimal C/N ratio 15:1 resulted in a N-larvae rate of 9% [21], while Pang et al. (2020b) reported that the optimal C/N ratio of 25:1 achieved a N-larvae rate of 22% [20]. However, both studies used high fiber materials to adjust the C/N ratio. Beesigamubama et al. (2021) added sawdust to brewer’s waste grain to obtain a C/N of 11, 15, 20, 25, and 30, while Pang et al. (2020b) added corncob to pig manure to achieve a C/N of 15, 20, 25, 30, and 35. Since the BSFL has difficulty digesting fiber-based materials without bacterial assistance [40,41], the addition of fiber carbon could hardly change the C/N of the digestible C and N. However, the total energy, porosity, and surface/volume ratio of the substrate could be altered. Therefore, the optimal conditions of C/N 15:1 [21] or 25:1 [20] could be highly derived from the balance of the total energy and porosity. Conversely, fiber-based materials were not used in the present study, and urea was applied as the material for C/N adjustment. The optimal C/N range of 21:1–16:1 for N-larvae conversion is generally the optimal range of digestible C and N ratios. The high N-larvae rates could be partially explained by the digestibility of the N source in the food waste and urea and partially attributed to the optimal C/N range, since C/N 21:1–16:1 and C/N 14:1–10:1 resulted in N-larvae rates of 64–75% and 35–46%, respectively.

### 3.2. Transcriptome Profiling of AMP Gene Family

The AMP gene family was defined as cecropin, attacin, defensin and lysozyme genes in the current study. Although several studies have reported other AMP types, such as diptericin, knottin-like protein, sarcotoxin, and stomoxyn [24,31]; however, these genes were not noted in the BSF reference genome (PRJEB37575) [37]. Furtherly, the lysozyme was considered as one of the components of AMP gene family, which was not common in the general scope of AMPs [24] whereas similar to Vogel et al. [31]. This is because: (1) lysozymes do contain the microbial inhibition activity, which belong to the definition of antimicrobial proteins; (2) there are 51 lysozyme genes expressed in the BSF transcriptome, which is much higher than that of 35 cecropin, 4 attacin, and 9 defensin genes, that highlights the importance of lysozymes in the microbial defense effect; and (3) the lysozymes are typically ≈140 amino acids and ≈15 kDa (PRJEB37575), the AMPs were therefore defined as the abbreviation of “antimicrobial proteins” instead of “antimicrobial peptides” in order to incorporate big molecules, such as lysozymes. Other than lysozymes, Vogel et al. [31] has also analyzed the peptidoglycan recognition proteins (PGRPs) and gram-negative bacteria binding proteins (GNBPs) in the study of BSF AMPs. However, since the PGRPs and GNBPs are mainly proteins acting in the immune signaling pathway, their contribution to the direct bacterial suppression could be limited, the current study therefore did not enclose the PGRPs or GNBPs in the AMP gene family. 

The SDS-PAGE analysis showed the current AMP extracts were crude protein extracts containing both AMP and non-AMP proteins with molecular weights distributed from 0–44.3 kDa. According to the reference genome (PRJEB37575) [37], the AMP molecular weights are mainly composed by ≈7 kDa (cecropin), ≈20 kDa (attacin), ≈10 kDa (defensin), and ≈15 kDa (lysozyme), which suggests the strong bands nearby the 14.3 kDa marker band were the AMPs. Although there were some non-AMP proteins remained in the crude extraction, such as bands nearby 20–44.3 kDa, the crude AMP extracts were not further purified, since the purification may alter the molecular structure of AMPs, even if some alternation may be not shown on the gel, and the relationship between the total transcriptome and the overall activity of AMPs could be affected. To be noted, the dissolving buffer for the lyophilized AMP extracts may affect the molecular structure of the crude AMPs. The sterile water solution resulted in molecular weights distributed from 0–44.3 kDa (Figure 1); however, the Tris-HCl buffer (100 mM, pH 8.0) resulted in molecular structure varying between 0–97.2 kDa (data not shown). Since the AMPs were mainly composed by ≈7–20 kDa proteins, the sterile water was therefore used to dissolve the lyophilized AMPs for the antimicrobial activity analysis in the current study.

To understand the C/N-dependent AMP activity and expression difference further, RNA-seq analysis was conducted on the C/N 21:1, 18:1, and 16:1 samples. C/N 14:1, 12:1, and 10:1 were not sequenced due to low performance in the N-larvae conversion and waste remediation. Among C/N 21:1, 18:1, and 16:1, the antimicrobial activity of AMP extracts could be ranked as follows: C/N18:1 > C/N16:1 > C/N21:1. The evidence were that C/N 18:1 and C/N 16:1 exhibited lower IC_50_ and MIC values than C/N 20:1, and C/N 18:1 showed a more enhanced inhibition zone than C/N 16:1 in the DIZ assay. Thus, it was interesting to understand what was the driving force of AMP activity difference among the C/N21:1, 18:1 and 16:1, the bacterial community or the larval transcriptome regulation. The bacterial community analysis revealed that there was limited difference of bacterial structure among the frass of C/N21:1, 18:1 and 16:1 (Figure 9). Therefore, the larval transcriptome regulation could be the main reason for the AMP activity difference.

The transcriptome reference-based assembly achieved good quality since the abundance of gene transcripts showed normal distribution in the TPM plotting, and the replicated samples exhibited high reproducibility (Figure 5). Among the C/N 21:1, 18:1, and 16:1 samples, the common trait of the AMP gene family was that the lysozymes were significantly more highly expressed than the cecropins, attacins, and defensins, that is, approximately 10^3^–10^4^ TPMs vs. 0–10^2^ TPMs (Figure 6). The differences were that C/N 18:1 and 16:1 significantly up-regulated lysozyme genes and down-regulated several cecropin, attacin, and defensin genes, compared with C/N 21:1 (Figure 6). Since the lysozyme genes were expressed approximately 10^2^ times more highly than the cecropin, defensin, and attacin genes, the regulation of lysozymes may have contributed more to the AMP activity difference. The lysozyme expression abundance ranked as C/N 18:1 > C/N 16:1 > C/N 21:1 based on the transcriptome profiling of lysozyme genes (Figure 6). Such ranking agreed with the antimicrobial activity of AMPs. The results suggest that the lysozymes were the main component in the AMP family, and the up-regulation of lysozyme expression contributed to the improvement in AMP antimicrobial activity. Out of 51 lysozyme genes, C/N 18:1 and C/N 16:1 up-regulated (*p* < 0.05) 14 and 12 genes compared with C/N 21:1, respectively, corresponding to the higher activity of AMPs.

Vogel et al. (2018) also conducted transcriptome research on the AMP gene family, but applied de novo analysis due to the limitation of the reference genome [31]. Vogel et al. (2018) also found that the lysozyme genes were more highly expressed than the cecropin, defensin, and attacin genes, which is consistent with the present study. However, the expression difference between the lysozyme and the remaining genes was −0.5–12.5 RPKM vs. −2.5–10 RPKM, and no relationship between the AMP expression level and antimicrobial activity was found. There is a high possibility that the uncertainty of gene identification and the lower accuracy of transcript quantification derived from de novo analysis introduced bias into the analysis, since less expression difference and lower gene numbers were found in Vogel et al. (2018)’s study than the present study. Transcriptome reference genome assembly may narrow the assembly error and facilitate the transcript quantitation and protein activity characterization. In addition, the high-quality chromosome-scale genome analysis and generous release of protein-coding gene annotation [37] were highly beneficial, since the present reference-based assembly would not be possible without such information, and the abundance comparison could also be biased or less accurate.

Although cecropins and defensins were widely extracted, purified, recombinantly expressed, and characterized in recent studies [24,25,26,30], lysozymes could be the main gene expressed in the AMP gene family other than cecropin and defensin, since the transcript difference was 10^3^–10^4^ TPM vs. 10^0^–10^2^ TPM. Notably, Park et al. (2015) and Choi et al. (2018) reported that there were difficulties in detecting and extracting defensin DLP4 or HP/F8 from the BSFL prior to *Staphylococcus aureus* or *Lactobacillus casei* stimulation, which indicates that the defensin protein was not highly expressed before the bacterial injection. Such findings agree with those of the present study, in that the BSFLs were harvested after food waste treatment without specific stimulation. Therefore, there is a high possibility that lysozymes were the main component of AMPs in the BSFL derived from waste treatment. Future studies could be conducted on the quantification, purification, and characterization of BSFL lysozymes.

### 3.3. Regulation of Entire Transcriptome

The GO enrichment network highlighted that gene functions related to hydrolase activity (acting on glycosyl bonds), muscle cell differentiation, and cytolysis were significantly up-regulated in C/N 18:1 than C/N 21:1 (Figure 7), and transcript comparison further confirmed these results, that 5 out of 27 hydrolase genes and 8–9 out of 23 muscle cell protein genes in C/N 18:1 and C/N 16:1 were found up-regulated than in C/N 21:1 (Figure 8). These results highlight the possibility that C/N 18:1 and 16:1 benefited from the additional supply of the N source for the synthesis of glycosyl hydrolase, muscle proteins, and lysozymes, which allowed for better utilization of the starch substrate, higher differentiation of muscle cells, and an improved immune defense system. The more connection of hydrolase activity than other gene functions in the GO network (Figure 7) indicates the significance of glycosyl hydrolase function, and also implies the adaptation of the BSFL to the starch-rich environment. Notably, a recent transcriptome analysis also highlighted the adaptation of the BSFL to the substrate. Bonelli et al. (2020) found that gene functions related with digestion and absorption were significantly regulated in the substrate alternation, that amylase was found significantly down-regulated in the substrate shift from a standard diet to a vegetable-based substrate [34]. The present food waste contained a relatively high amount of starch, and the beneficial C/N ratio of 18:1 and 16:1 may facilitate the transcriptome regulation for the enhancement of glycosyl hydrolase synthesis and thereby resulting in an improved waste transformation and AMP production. Moreover, C/N 18:1 and 16:1 were also found to significantly down-regulate the heat shock protein genes than C/N 21:1 (Figure 7 and Figure 8), which suggests that the BSFLs under C/N 18:1 and 16:1 were less stressed.

### 3.4. Application of Waste Derived Antimicrobial Proteins

In the present study, BSFLs were demonstrated to be a promising natural source of AMPs; the food waste C/N ratio did not alter the antimicrobial spectrum of AMPs, but did interact with the activity and expression of AMPs. Adjusting the C/N ratio to the range of 18:1–16:1 obtained improved antimicrobial activity of AMPs compared with C/N 21:1. The MIC of C/N 18:1 and 16:1 achieved 2.5–4.0 mg/mL against the five test pathogens, which was comparable to the MIC of BSFLs derived from needling and bacterial joint infection, i.e., 1.0–10.0 mg/mL [27,29]. Considering the practicability and quantity outcome, the C/N ratio adjustment is highly recommended in the BSFL farming for food waste treatment targeting improved AMP production.

In the comparison of AMPs with animal antibiotic SMT, the BSFL AMPs were comparable in the IC_50_ and MIC assay, but less efficient in the DIZ test. These results could be attributed to two possible reasons. Firstly, the different molecular size and hydrophilicity may interact with the inhibition zone test [42,43], and the AMPs resulted in bigger molecular size and lower diffusion speed than the SMT, which may contribute to the smaller inhibition zones. Secondly, the BSFL AMPs may inhibit pathogen cells but not fully kill the pathogens [24,44], and the semi-solid medium was more sensitive than the broth medium for the detection of bacterial regrowth, so that the AMPs resulted in better performance in the liquid IC_50_ and MIC test than the semi-solid DIZ test due to the regrowth of the inhibited pathogen cell. Evidently, increasing doses of AMPs may improve the pathogen inhibition effect, and further study could focus on the AMP antimicrobial effect both in vitro and animal in vivo, in order to provide deeper understanding of the AMP defense effect for animals, and support the application of waste derived AMPs for the replacement of animal antibiotics and defense against animal disease outbreak.

## 4. Materials and Methods

### 4.1. Strains and Reagents

Five pathogenic strains, i.e., *Escherichia coli O157:H7* (NCTC12900, EC), *Salmonella enterica* serovar typhimurium (ATCC14028, SE), *Staphylococcus aureus* (ATCC43300, SA), *Listeria Monocytogenes* (CMCC(B)54002, LM), and *Shigella Castellani* (CMCC(B)51592 SC) were purchased from Luwei Technology Co., Ltd. (Shanghai, China). Chemicals acting as analytical reagents, e.g., urea, Tris-HCl, acetic acid, Na_2_EDTA, NaCl, KCl, Na_2_HPO_4_, KH_2_PO_4_, coomassie brilliant blue R-250, sulfamonomethoxine, etc., were purchased from Aladdin Biochemical Technology Co., Ltd. (Shanghai, China). A BCA assay kit (C503051) for protein quantification and tryptone soy broth (TSB) medium were purchased from Sangon Biotech Co., Ltd. (Shanghai, China).

### 4.2. Food Waste Treatment

The experiment was carried out in Jul–Aug, 2020 in the laboratories of Dalian University of Technology, Panjin Campus, Panjin, China, North latitude 40°41′20.26″, East longitude 122°7′15.17″. Eggs of the black soldier fly, *Hermetia illucens,* were purchased from Langhao Environmental Technology Co., Ltd. (Nanjing, China), and hatched in a matrix containing soybean meal: corn flour: wheat bran in a 6:3:1 ratio with 65% of water content. Approximately 25 g eggs were hatched by 500 g materials at 25 °C for 5–6 days until small larvae were visible. The small larvae were separated by a 2 mm sieve and used for the food waste treatment. The food waste was obtained from the university canteen, namely cooked food leftovers, e.g., rice, steam buns, noodles, vegetables, tofu, eggs, meats, and other crumbs. The collected food waste was thoroughly mixed, and determined for moisture and C, N content in triplicate. The moisture content was found as ≈70%, and the total carbon (TC) and total nitrogen (TN) were determined to be 46.98% and 2.26%, respectively, which led the initial C/N to be ≈21:1. Then, 0.228 g, 0.437 g, 0.707 g, 1.066 g, and 1.569 g urea were added to every 100 g (wet wt) food waste to form C/N 18:1, C/N 16:1, C/N 14:1, C/N 12:1, and C/N 10:1 substrates, and the food waste without urea addition was used as the blank control, C/N 21:1. The adjusted 500 g food waste and 1200 individuals of larvae were loaded into the experimental boxes with triplicate sets for each C/N condition (n = 3). A total of 10 larvae were averaged at 0.0059 g (n = 3), and 1200 larvae were obtained by weighting 0.708 g of larvae. The boxes were 4 L transparent plastic boxes with approximately 20 holes (4 mm diameter) drilled in the box cover. The treatment was carried out for 10 d, with room temperature in a range of 25–32 °C and daily light period of ≈13–14 h. Upon d 10, larvae and frass were separated manually, weighed respectively, and stored at −20 °C.

### 4.3. Chemical Properties and Bioconversion Efficiency

The food waste, larvae, and frass samples were determined for moisture, TC, and TN. Approximately 5–10 g of the sample were dried at 65 °C to a constant weight (>16 h) to achieve the moisture content (Liu et al., 2018). The dried samples were ground into fine powder, and subjected to a Vario EL Cube Elemental Analyzer (Elementar Analysensysteme GmbH, Hanau, Germany) for the TC and TN determination. The frass pH was measured as a 1:10 (*w/v*) water soluble extract [45] using a FE38 pH meter (Mettler-Toledo GmbH, Zurich, Switzerland). The bioconversion efficiency was estimated using following equations:

(1) $$\mathrm{Waste}\;\mathrm{reduction}\;\mathrm{rate}\;\left(\%,\;\mathrm{WR}\right)=\frac{W-F}{W}\times 100\%$$

(2) $$\mathrm{Larvae}\;\mathrm{yield}\;\left(\%,\;\mathrm{LY}\right)=\frac{L}{W}\times 100\%$$

(3) W= F+L+S

(4) $$W_{C}={\;F}_{C}+L_{C}+S_{C}$$

(5) $$W_{N}={\;F}_{N}+L_{N}+S_{N}$$

where W, F, L, and S are the total dry mass of the food waste (Day 0), frass (Day 10), larvae (Day 10), and gaseous loss (Day 10); W_C_, F_C_, L_C_, and S_C_ are the total carbon mass of the food waste, frass, larvae, and gaseous loss; and W_N_, F_N_, L_N_, and S_N_ are the total nitrogen mass of the food waste, frass, larvae, and gaseous loss. All weights were measured in g with dry matter basis.

### 4.4. Protein Extraction and Quantification

The frozen BSFL were thawed to the room temperature, cleaned with bleach (2% NaClO) and sterile water, dried with tissue papers prior to the extraction. The extraction buffer was prepared using 10% acetic acid and 0.01 mol/L Na_2_EDTA water solution following Lee et al. (2020b) with modifications [29]. The BSFL were mixed with the buffer in a 1:10 (*w*/*v*) ratio and homogenized in a JM-L50 colloid mill (Huawei Co., Ltd., Wenzhou, China) for 2 min and repeated for 3 times. The homogenate was centrifuged by a TD5A-WS centrifuge (Xiangyi Laboratory Instrument Co., Ltd., Changsha, China) at 3500 rpm for 25min with supernatant collected and measured for volume and protein concentration using a commercial BCA kit (Sangon). The AMP extraction rate (ER) was calculated as C × V/A, where C, V, and A represent the supernatant protein concentration, supernatant volume, and larval weight used for the extraction on dry matter basis. The AMP solution was further freeze-dried for the evaporation of acetic acid and stored at −20 °C. Prior to the SDS-polyacrylamide gel electrophoresis (SDS-PAGE), the lyophilized AMP powder was resuspended in double distilled (d.d.) H_2_O, treated by ultrasonication, centrifuged at 10,000 rpm for 2 min and quantified by a BCA kit. Protein marker 3597A (Takara Biotechnology Co., Ltd., Dalian, China) and 40 μg AMP protein of each sample were loaded onto the SDS-PAGE gel (5% concentrating and 12% separating gel) and electrophoresed at 90 V for 45 min and 120 V for 1.5 h followed by coomassie brilliant blue staining and digital photographing.

### 4.5. Assay of Antimicrobial Activities

The AMP extracts were examined for the half inhibition concentration (IC_50_), minimum inhibition concentration (MIC), and diameter of inhibition zone (DIZ) assays, corresponding to the 5 pathogenic strains (EC, SE, SA, LM, SC), respectively. The IC_50_ and MIC assays were based on the inhibition test according to Choi et al. (2018). Briefly, the bacterial strains were cultured overnight in TSB medium and diluted to the concentration of ≈1.0 × 10^5^ CFU/mL with phosphate buffered saline (1 × PBS, pH7.4). The lyophilized AMP extracts were resolved in d.d. H_2_O and quantified by a BCA kit, and made in a 2-fold serial dilution with 1 × PBS buffer (start concentration 8.0 mg/mL). The inhibition assay was performed by mixing the bacterial solution (10^5^ CFU/mL), the AMP serial dilution, and the TSB medium in a 1:2:1 volume ratio associated with incubation at 37 °C for 12 h. The 1 × PBS was used to replace AMP solution to form the blank control. The incubations were measured for the optical density (OD) under 600 nm at 0 h (OD^0h^) and 12 h (OD^12h^), respectively, using the Multiskan MK3 microplate reader (Thermo Scientific, Wilmington, DE, USA). The inhibition rates were determined in triplicate with the formula as follows:

(6) $$\mathrm{Inhibition}\;\mathrm{Rate}\;(\%)=(1-\frac{{\mathrm{OD}}_{\mathrm{sample}}^{12h}-{\mathrm{OD}}_{\mathrm{sample}}^{0h}}{{\mathrm{OD}}_{\mathrm{control}}^{12h}-{\mathrm{OD}}_{\mathrm{control}}^{0h}})\times 100\%$$

The inhibition curves were built using GraphPad Prism version 9.0.0 (GraphPad InStat Software, Inc., San Diego, CA, USA) with IC_50_ and 95% confidence interval (CI) estimated by the non-linear regression model of Y = 100 × X^h^/(EC_50_^h^ + X^h^). The MIC assays were also based on the inhibition assay in triplicate, with the AMP concentration adjusted to 10.5, 10.0, 9.5, 9.0, 8.5 mg/mL for C/N 21:1 and 5.0, 4.5, 4.0, 3.5, 3.0, 2.5, 2.0 mg/mL for C/N 18:1-C/N 10:1. The MICs were defined as the minimum AMP concentrations achieving an inhibition rate >95% (Park et al., 2015). Additionally, the antibiotic sulfamonomethoxine (SMT) was used as a positive control in both the IC_50_ and MIC assay to compare with AMP extracts.

The DIZ assay was conducted following Choi et al. (2018) with modifications [26]. Briefly, resolved and quantified AMP solutions were prepared to 12 mg/mL, and a positive control of SMT was prepared to 3 mg/mL for the assay. Solid plates were made with 1.5% TSB and 1.5% agar. The bacterial cultures were prepared to ≈1.0 × 10^5^ CFU/mL in 1 × PBS buffer. The bacterial solutions were spread onto the plate with 1 mL aliquot and withdrew afterwards. The plates were air dried for 30 min at room temperature and loaded with sterile 7.5 mm-diameter Oxford cups. A total of 100 μL of AMP or SMT solutions were added into the cups and incubated at 37 °C for 12–16 h (n = 3). The inhibition zones were observed, and the DIZs were measured and photographed after the incubation.

### 4.6. Transcriptome Analysis

The larvae samples of C/N 21:1, 18:1, and 16:1 (Day 10) were processed for the transcriptome sequencing with the MGISEQ-2000 High-throughput Sequencing Set (Beijing Genomics Institute, BGI, Shenzhen, China) in Sangon Biotech Co., Ltd. (Shanghai, China). Briefly, the frozen larvae samples were stored at −80 °C and shipped to Sangon in dry ice. Triplicate individual larva samples were used for the RNA extraction, transcriptome library construction, and high-throughput sequencing according to the Sangon standard protocol. The sequenced raw data were examined, spliced, and assembled according to the reference genome PRJEB37575 (NCBI database). Transcripts belonging to the same gene were combined and recorded as reads (X_i_). To normalize reads’ data, the TPM (transcript per million) concept was introduced and calculated as follows:

(7) $${\mathrm{TPM}}_{i}=\frac{X_{i}}{L_{i}}\times \frac{1}{\sum_{j}\frac{X_{j}}{L_{j}}}\times 10^{6}$$

where X_i_ is the reads of gene I; L_i_ is the length (Kb) of gene I; X_j_ is the reads of gene j; L_j_ is the length (Kb) of gene j; and Σ_j_ (X_j_/L_j_) is the sum of X_j_/L_j_ over the entire transcriptome. Thus, the TPM represents the proportion of one-millionth of a certain gene’s mRNA in the total mRNA pool, which allows for the expression comparison among genes across samples. The TPM data of the AMP gene family, i.e., cecropin, attacin, defensin, and lysozyme, were extracted from the transcriptome data and compared statistically. Further, the AMP gene family was analyzed using Mega-X software [46] for the construction of the UPGMA evolutionary tree based on their protein sequences.

To compare the entire transcriptome further, anosim analysis was engaged using the vegan package in R 3.4.1 software (R core team, 2021) to compare the transcript abundance among C/N 21:1, 18:1, and 16:1. Further, GO (Gene Ontology) enrichment analysis was utilized for the comparison of gene functions. Briefly, all genes in the transcriptome were annotated with GO terms (biological process, cellular component, or molecular function) according to the GO database (http://www.geeontology.org, accessed on 18 March 2022). Genes with the same GO terms were combined, and significantly regulated GO terms were identified using the DESeq package in the R 3.4.1 software. The top 10 regulated GO terms (based on *p* value sorting) associated with the related genes were extracted and used for the construction of the GO enrichment network with the igraph package in the R 3.4.1. The relationship of significantly regulated genes and gene functions were identified through GO network plotting. The RNA-seq data were deposited in the NCBI Sequence Read Archive (http://www.ncbi.nlm.nih.gov/sra, accessed on 18 March 2022) under BioProject number PRJNA788971.

### 4.7. Bacterail Community Analyses

The frass samples of C/N21:1, 18:1, and 16:1 as well as d 0 food waste were detected for the bacterial community targeting 16S rRNA V3-V4 region with Illumina MiSeq platform (Illumina Inc., CA, USA) in Sangon Biotech Co, Ltd. (Shanghai, China) following Sangon standard protocols. Briefly, the frozen frass samples were sent to Sangon in dry ice, and extracted for the genomic DNA. The genomic DNA was amplified from the V3-V4 region of 16S rRNA gene and constructed for a bacterial community library and applied to the Illumina Miseq Genome Sequencer according to manufacturer’s protocols. The sequenced results were recorded as reads, which were classified into OUTs (operational taxonomic units) and further classified into taxonomy groups according to the blast results of OTUs against the RDP 16S rRNA database (http://rdp.cme.msu.edu/misc/resources.jsp, accessed on 18 March 2022). The composition percentages of the phylum, order, and genus level were calculated and presented as color columns. Each sample of C/N21:1, 18:1, and 16:1 as well as d 0 food waste samples were examined in triplicate with abundance data averaged and compared among the groups. The composition differences were compared with the R 3.4.1 software vegan package anoism assay. The MiSeq sequencing data have been deposited in the NCBI Sequence Read Archive (http://www.ncbi.nlm.nih.gov/sra, accessed on 18 March 2022) under BioProject number PRJNA788678.

### 4.8. Statistical Analyses

The R 3.4.1 software was engaged with the statistical analysis [47]. The chemical and bioconversion differences among C/N groups were examined with the multcomp package using one-way variance analysis (ANOVA), associated with the TukeyHSD assay for pairwise comparison of means. The gene expression differences were examined with the DESeq package to calculate the mean TPMs, log_2_ Fold Changes, *p* values, and *Q* values (adjusted *p* value). Statistical significance was defined as *p* < 0.05.

## 5. Conclusions

Black soldier fly larvae derived from food waste treatment were demonstrated to be a promising natural source of antimicrobial proteins. The substrate C/N ratio interacted with both the element bioconversion efficiency and the expression of AMPs. The C-larvae ratio obtained 27.0–35.5%, being higher than previous studies due to the high energy of food waste substrate, the high digestibility of the C source, and the feasibility of the larvae/waste ratio. The N-larvae ratio achieved 35.0–75.0%, especially for C/N 21:1–16:1, being 63.5–75.0%, which was attributed to the high digestibility of the N source and optimal C/N ratios. The food waste derived AMPs possessed a wide spectrum of antimicrobial ability. The C/N ratio did not alter the antimicrobial spectrum, but did change the activities of AMPs, with C/N 21:1 being significantly lower than C/N 18:1–10:1. The lysozyme genes were found to be significantly more highly expressed than the cecropin, defensin, and attacin genes in the AMP gene family. Out of 51 lysozyme genes, C/N 18:1 and C/N 16:1 up-regulated (*p* < 0.05) 14 and 12 genes compared with C/N 21:1, respectively, corresponding to the higher activity of AMPs. Further, C/N 18:1 and 16:1 significantly up-regulated gene functions of hydrolase activity (acting on glycosyl bonds), muscle cell differentiation, and cytolysis compared with C/N 21:1, which likely enhanced the starch digestion, the muscle cell development, and the immune defense system. The frass bacterial community was not the driving force of AMP activity difference, whereas the larval transcriptome regulation was. The waste derived AMPs, especially from C/N 18:1 and 16:1, possessed comparable antimicrobial activity as the specifically stimulated AMPs, which are recommended for the agriculture application for the defense against pathogenic infection. The C/N ratio manipulation is highly recommended for the BSFL farming targeting improved AMP production and high efficiency of food waste management.

## Figures and Tables

**Table 1:** The bioconversion performance and AMP extraction rate of BSFL under different C/Ns.

Groups	WR (%)	LY (%)	C_protein_ (mg/mL)	ER (%)
C/N 21:1	87.9 ± 1.5c	26.5 ± 1.2	6.73 ± 0.39	15.7 ± 1.13
C/N 18:1	86.6 ± 1.7c	27.8 ± 3.2	7.29 ± 1.14	17.0 ± 2.88
C/N 16:1	87.1 ± 0.8c	30.6 ± 1.3	6.92 ± 1.09	16.8 ± 2.24
C/N 14:1	82.6 ± 1.2b	25.1 ± 4.1	6.71 ± 1.37	15.9 ± 3.51
C/N 12:1	79.5 ± 0.9b	25.7 ± 2.1	6.16 ± 0.75	14.8 ± 1.45
C/N 10:1	69.0 ± 2.1a	24.1 ± 0.9	5.66 ± 0.92	13.5 ± 2.42
*p*	<0.001	0.177	0.646	0.714

Note: WR, waste reduction rate; LY, larvae yield; C_protein_, the extracted protein concentration; ER, the AMP extraction rate. Data were presented as mean ± SD, n = 3. Statistical analyses were made based on columns with *p* values labeled at the bottom, and values in the same column with different superscript letters were significantly different.

## Data Availability

The data presented in this study are openly available in FigShare at https://doi.org/10.6084/m9.figshare.17194964.v1 (accessed on 18 March 2022).
